# AI-Driven Injury Reporting in Pediatric Emergency Departments

**DOI:** 10.1001/jamanetworkopen.2025.24154

**Published:** 2025-07-31

**Authors:** Devin Singh, Alper Celik, Evangeline W. J. Zhang, Eric Liu, Daniel Rosenfield

**Affiliations:** 1 Division of Pediatric Emergency Medicine, Hospital for Sick Children (SickKids), Toronto, Ontario, Canada; 2 Department of Pediatrics, University of Toronto, Toronto, Ontario, Canada; 3 Center for Computational Medicine, SickKids, Toronto, Ontario, Canada; 4 Schulich School of Medicine and Dentistry, University of Western Ontario, Canada; 5 Department of Computer Science, University of Toronto, Toronto, Ontario, Canada

## Abstract

**Importance:**

Injury is a leading cause of morbidity and mortality among children worldwide. Prevention strategies rely on timely and accurate injury surveillance. Many national programs, including the Canadian Hospitals Injury Reporting and Prevention Program (CHIRPP), depend on manual review of emergency department (ED) medical records to track injury trends. Rising ED volumes have strained manual processes, delaying data analysis and compromising early detection of public health risks.

**Objective:**

To evaluate whether natural language processing (NLP) transformer models can automate injury case detection in ED medical records, improving CHIRPP workflow efficiency while maintaining high sensitivity.

**Design, Setting, and Participants:**

Prognostic study of ED visits from January 1, 2017, to December 31, 2023, at The Hospital for Sick Children, a high-volume tertiary pediatric referral center in Toronto, Canada, and a core CHIRPP site. The dataset included pediatric ED visits across all age groups. All medical records were labeled as requiring or not requiring CHIRPP reporting, with no exclusions. Two transformer-based NLP models, DistilBERT-base-uncased (model 1) and BERT-large-uncased (model 2), were fine tuned using supervised learning to classify medical records as CHIRPP-reportable or not.

**Exposure:**

Application of fine-tuned NLP transformer models to routine ED visit data to automate classification of injury-related cases for surveillance reporting.

**Main Outcomes and Measures:**

Outcomes included true positive rate (TPR), true negative rate (TNR), false positive rate (FPR), false negative rate (FNR), area under the receiver operating characteristic curve (AUROC), and area under the precision-recall curve (AUPRC). Additional outcomes included the proportion of medical records requiring manual review to achieve 90% sensitivity.

**Results:**

Among 217 173 pediatric ED visits across all age groups, model 1 achieved an AUROC of 0.983, AUPRC of 0.932, TPR of 0.90, TNR of 0.99, FPR of 0.014, and FNR of 0.10. Model 2 showed similar performance with an AUROC of 0.983, AUPRC of 0.931, TPR of 0.90, TNR of 0.99, FPR of 0.012, and FNR of 0.09. Both models identified 90% of injury cases while reducing manual medical record review from 100% to 17% of ED visits.

**Conclusions and Relevance:**

NLP transformer models accurately automated detecting injury cases in ED patient medical records, with the potential of enabling real-time injury surveillance monitoring.

## Key Points

**Question:**

Can natural language processing (NLP) models automate detecting injury cases in emergency department patient medical records to improve the efficiency of injury reporting and surveillance programs?

**Findings:**

In this prognostic study of 217 173 emergency department visits at The Hospital for Sick Children, NLP models identified 90% of injury cases and reduced manual medical record review from 100% of patient medical records to only 17%, representing an 83% reduction.

**Meaning:**

These findings suggest natural language processing models can effectively automate injury case detection in emergency department patient medical records, substantially improving the efficiency of injury surveillance reporting.

## Teaser

This prognostic study evaluates whether natural language processing transformer models can automate injury case detection in emergency department medical records.

## Introduction

Injury is the most common cause of morbidity and mortality among children outside of the neonatal period.^1^ Around the world, injury prevention initiatives depend on robust data collection, interpretation, and reporting, so that underlying causes can be addressed and prevented in the future.^2^ Without timely, accurate, and actionable data, there is no way to identify threats, monitor progress, or measure success. Around the world, various injury surveillance systems exist, such as the National Electronic Injury Surveillance System in the US, or the All Wales Injury Surveillance System in the UK.^3,4^ Historically, these systems relied heavily on manual data entry and interpretation. In this study, we describe a Canadian sentinel event–based injury surveillance system and the local use of natural language processing to automate data collection and interpretation.

The Canadian Hospitals Injury Reporting and Prevention Program (CHIRPP) is a national injury and poisoning surveillance initiative that tracks patients that present to emergency departments (EDs) across Canada. CHIRPP aims for early detection of epidemiological trends in injuries to guide public health decision-making, as most injuries and poisonings are preventable.^5^ Patient encounters that qualify for CHIRPP are deidentified and have information on circumstances surrounding the injury uploaded to a secure online database on the Canadian Network for Public Health Intelligence. For CHIRPP to optimally improve public safety, rapid reporting of injury data is required, which is dependent on effective methods for patient screening and subsequent data processing.

Since 2010, Ontario ED patient volumes have been increasing at greater than twice the rate of population growth.^6^ Pediatric emergency departments have also seen similar rates of volume increases over the past several years.^7,8^ As a result, national health surveillance initiatives such as CHIRPP have been experiencing increasing workloads and delays in case reporting. The ED at The Hospital for Sick Children (SickKids) in Toronto is a CHIRPP center with one of the highest pediatric patient volumes in Canada, seeing over 80 000 children annually. This poses a challenge that increases the backlog of patient encounters, causing significant delays in injury reporting due to lengthening the time gap between time of patient injury and reporting and/or analysis. The previous workflow for CHIRPP at SickKids involved human workers manually reading all of the physician or nursing notes in the electronic medical record to identify patient encounters that qualify for injury reporting. Data such as patient demographics, geographic location of injury, preceding events, and pertinent past medical history are populated onto a data entry form and subsequently reported to the Public Health Agency of Canada. The COVID pandemic added to significant barriers to data entry as paper forms previously given to families were removed due to infection control measures. Overall, due to the increasing patient population, complexity of injuries and presentations, and pandemic-related restrictions, the data-input backlog was approximately 3 years, undermining the effectiveness of public health interventions and limiting the utility of the data collected. To respond to these challenges, a new artificial intelligence (AI)–augmented workflow was created and its performance evaluated in this study.

Natural language processing (NLP) is a machine learning technique that analyzes free text (unstructured) data and has demonstrated efficacy in predicting patient disposition based on triage nursing notes in the context of the ED.^9^ Deleger et al^10^ showed NLP decision-making based on triage notes, provider notes, labs, and vitals to be equivalent to that of physicians for diagnosing appendicitis in risk-stratified patients presenting to a pediatric ED.^10^ NLP has also shown efficacy in identifying abuse in pediatric presentations to the ED using both free text radiology reports and quantitative data.^11^ While existing literature has effectively applied NLP for the identification of specific injuries and diagnoses, wide scale injury screening by NLP has not been previously well documented. In this study, we demonstrate an application of NLP transformer models in estimating patient injury and inclusion in the CHIRPP national injury surveillance program based on free-text practitioner notes from a pediatric emergency department in a tertiary care center. We hypothesize that implementation of NLP will streamline the patient screening step of the CHIRPP workflow at SickKids. Rather than having humans manually review 100% of patient medical records in the ED, we aim to use NLP to automate the screening of patient medical records, dramatically reduce the number of manual medical records to be reviewed, and reduce the time delays associated with traditional injury reporting programs.

## Methods

This work was completed with research ethics board approval from SickKids and with a waiver of consent for retrospective, deidentified, secondary use of data. Reporting was completed in accordance with the Transparent Reporting of a Multivariable Prediction Model for Individual Prognosis or Diagnosis-AI (TRIPOD-AI) reporting guideline for prediction model studies.^12^

### Dataset and Labeling

**Figure 1.**
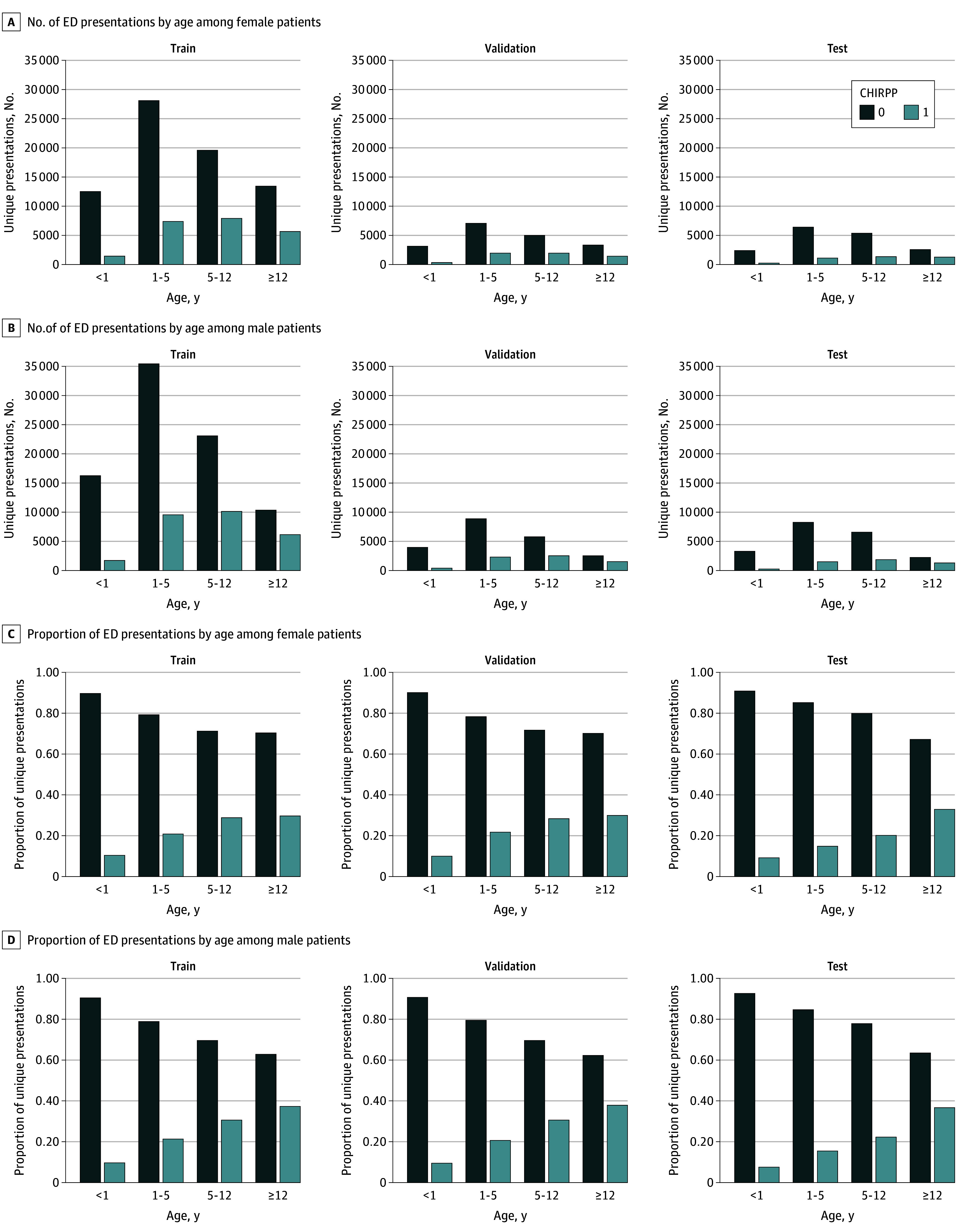
Age and Biological Sex Distribution of Emergency Department (ED) Patient Presentations Distribution of Canadian Hospitals Injury Reporting and Prevention Program (CHIRPP)–positive and CHIRPP-negative cases by age and sex assigned in the health record, within the train, validate, and held-out test datasets.

Electronic health record data were obtained for all ED visits occurring between October 2018 and January 2023 at SickKids. Data elements included the following: free text clinical notes, chief complaints, ED diagnosis, and date of arrival. These data were hand-labeled by a human for whether or not patients satisfied the injury reporting criteria for CHIRPP (ie, CHIRPP-positive vs CHIRPP-negative). Our dataset has been compiled by our CHIRPP coordinator (E.W.J.Z.). This individual has a graduate degree in data analysis and interpretation, and is tasked with reviewing all ED data for injuries and poisonings. They have been employed in the role for over 10 years. Our total dataset contained 217 173 individual patient visits to the ED at SickKids. Our training and validation set consisted of 203 625 patient visits with 50 488 (24.8%) cases being CHIRPP-positive. Our held-out test set contained 13 548 patient visits, of which 2175 (16%) were CHIRPP-positive (Figure 1).

### Data Preprocessing

**Figure 2.**
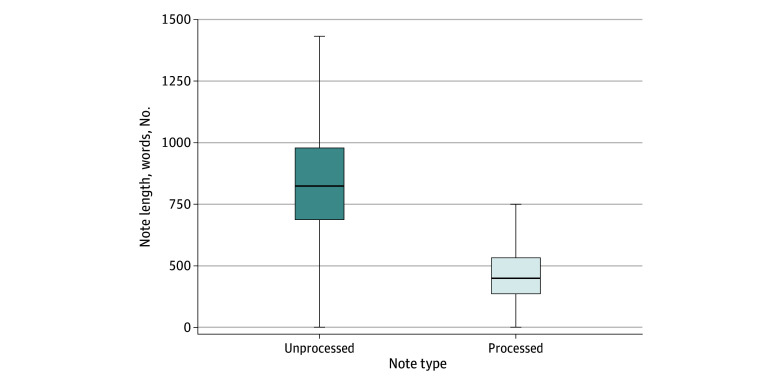
Note Length Before and After Clinical Note Preprocessing This figure shows the significant reduction in note length after targeted preprocessing techniques. Full emergency department physician notes (dark blue) contain information that is not required for efficient classification of injury cases. Automating removal of these sections in a context-aware manner maintains all the relevant information while reducing the note size. This allows for the most important information to fit within the token limit of the transformer models, reduces noise within the data, and adds computational and cost efficiencies when running models at scale. Boxes represent the middle 50th percentile of the distribution of the data. Whiskers represent the 5th percentile to the 95th percentile. Horizontal lines show the 50th percentile.

Data processing and subsequent machine learning model development and validation occurred from January 2022 to November 2024. Most transformer models use a fixed document size usually in the range of hundreds to low thousands.^13,14^ Our concatenated ED notes usually exceeded this limit, causing notes to be truncated to be used by the model. To overcome this limitation, we used spaCy^15^ and medspacy^16^ to search and extract highly predictive sections of the free text clinical notes, such as the patient history of presenting illness, diagnoses, and physician assessments. We used the same method to exclude sections like laboratory results and vital signs. This preprocessing step reduces the median and mean note size from 752 to 292 and 802 to 322 words, respectively, well within the 512 tokens that can be processed at 1 time for the models we chose (Figure 2). This also added to the feasibility of model training and iterative improvements by reducing compute power and time. To further process our clinical notes, we manually generated a dictionary of common abbreviations to convert medical abbreviations to longer form text.

### Model Fine Tuning for Classification

For our binary classification models we selected several transformer models for our initial testing from the Hugging Face model repository.^17^ We chose to mainly focus on encoder-based transformer models since our task focused on interpretation of model embeddings and did not require text or image generation. Our model selection was also targeted to relatively lean models when compared with large language models that contain billions of parameters, are expensive to train and host, and may involve data privacy and security concerns when hosted on public clouds. Our model training was done on-premise at SickKids using high performance computing clusters and a single NVIDIA A100 GPU for 10 epochs with a batch size of 120 and a learning rate of 1e-5 with an AdamW optimizer. To further minimize noise introduced by small minibatches we used 4 gradient accumulation steps. Before model training, all the training and validation data were randomized separately for positive and negative cases and reshuffled before train and validation split. We used 70:30 train and validation split and the best model was chosen automatically based on area under the receiver operating characteristic curve (AUROC) on the validation set. Within the selected models, DistilBERT-base-uncased (model 1)^14^ and BERT-base-large-uncased (model 2)^13^ showed the best performance. To generate our final outcome metrics, we used a held-out test set of the most recent manually labeled notes (13 548 notes).

### Statistical Analysis

Model performance was assessed using scikit-learn version 1.6.0 to compute the following outcome metrics: AUROC, area under the precision-recall curve (AUPRC), true positive rate, true negative rate, false positive rate, and false negative rate. All visualizations were done using R version 4.4.3 (R Project for Statistical Computing) and the packages ggplot2 version 3.5.1 and patchwork version 1.2.0. For most surveillance systems, a high true positive rate (sensitivity) is desired since missing cases may defeat the purpose of monitoring what can often be a low frequency event. As such, we tailored our algorithms by selecting a model operating threshold to yield a true positive rate of 0.90 and subsequently generated all other threshold-dependent outcome metrics.

## Results

After data preprocessing, our total dataset contained 217 173 individual patient visits to the ED at SickKids. Our training and validation set consisted of 203 625 patient visits with 50 488 cases (24.8%) being CHIRPP-positive. Our held-out test set contained 13 548 patient visits, of which 2175 (16%) were CHIRPP-positive with associated demographic details (Figure 1).

**Figure 3.**
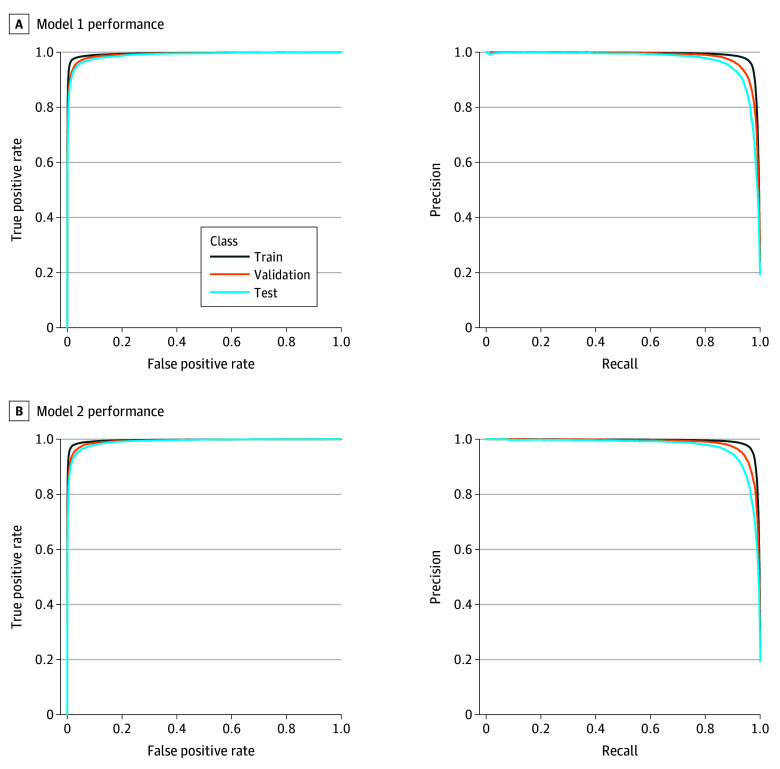
Model Performance A, Area under the receiver operating characteristic curve (AUROC) and area under the precision-recall curve (AUPRC) for model 1. B, AUROC and AUPRC for the model 2.

Both models demonstrated strong performance including a true positive rate of 0.9 and true negative rate of 0.99 for both model 1 and model 2. False positive rates of 0.014 (model 1) and 0.012 (model 2) and false negative rates of 0.1 (model 1) and 0.09 (model 2) were also obtained as outlined in the Table. The AUROC and AUPRC for model 1 and model 2 were 0.983, 0.932, and 0.983, 0.931, respectively, on our held-out test set, as seen in Figure 3.

**Table.** Outcome Metrics Generated Using a Held Out Test Set

Metric	DistilBERT	BERT-large
Area under the precision recall curve (95% CI)	0.932 (0.9288-0.9352)	0.931 (0.9282-0.9338)
Area under the receiver operating characteristic curve (95% CI)	0.983 (0.982-0.984)	0.983 (0.982-0.984)
True positive rate^a^	0.90	0.90
False positive rate (95% CI)	0.014 (0.0124-0.0156)	0.012 (0.0104-0.0136)
True negative rate (95% CI)	0.99 (0.9884-0.9916)	0.99 (0.9884-0.9916)
False negative rate (95% CI)	0.1 (0.05-0.15)	0.09 (0.0844-0.0956)

^a^ The model operating and decision threshold was set to yield a true positive rate of 0.90 to meet the performance standards of the Canadian Hospitals Injury Reporting and Prevention Program.

**Figure 4.**
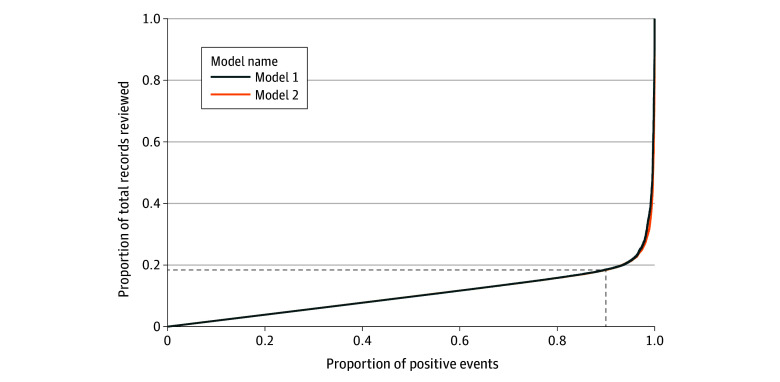
Trade-Off Between Model True Positive Rate and Manual Medical Record Review The trade-off between the number of positive injury cases that can be detected by both classification models compared with the corresponding fraction of total emergency department medical records that are required for manual review.

The main goal of this model is to reduce the total number of patient medical records required for manual human review while still capturing a high number of injury cases for reporting. To this end, to capture 90% of injury cases (ie, true positive rate of 0.90), our team will only need to process 17% of total ED patient medical records for model 1 and 16% of patient medical records for model 2. Previously, all 100% of patient medical records required manual review. With our models, manual work related to patient medical record screening can be reduced by approximately 83%. This leads to a significant reduction in time associated with manual human labor. Figure 4 demonstrates the model trade-off between the fraction of positive injury cases that can be captured by the model vs the number of total ED medical records that are required for manual human review.

For example, in 2019, there were 37 037 CHIRPP patients, out of a total of 77 677 total ED visits. Screening each medical record manually to determine CHIRPP eligibility by a very experienced coordinator takes approximately 20 seconds per medical record (approximately 432 hours of work), and thus by reducing the number of medical records screened by 64 472 medical records, our team has saved over 358 hours of screening per year, or more than 10 weeks of full-time work (working a 35-hour work week), resulting in time savings. This does not take into account natural human fatigue and breaks during the workday, which would further add to the time-savings estimates.

## Discussion

Increasing annual patient volumes in EDs have made it extremely difficult to identify which patients fit a specific set of criteria for inclusion into surveillance and reporting programs such as CHIRPP.^7,8^ Manual workloads and related reporting delays for CHIRPP and other public health surveillance programs have the potential to be greatly reduced with high sensitivity and specificity as demonstrated by our fine-tuned-models in Figure 3. Compared with the traditional method of humans manually screening free text clinical notes to determine if a patient met the inclusion criteria for injury reporting, transformer models can automate this process and expedite reporting by removing patients ruled out by the algorithm, resulting in a reduced set of patient medical records for human review.

Recent studies have demonstrated the utility of machine learning and NLP to process free-text clinical notes and automate injury classification and surveillance in emergency settings.^18,19,20^ The current study advances this field by applying transformer-based models to injury classification and has the potential for higher generalizability at scale due to the ability to learn contextual language patterns directly from raw text, reducing reliance on manual feature engineering.

While high-traffic emergency departments that participate in the CHIRPP program can benefit from adding this automation step to their workflow, the results demonstrated in this study show promise for applications in many other surveillance programs that seek to categorize patients based on a set of criteria. There is also an opportunity to add increasing amounts of automation to the reporting process overall, such as automating summarization of cases. Emerging large language models show great promise in facilitating automated summaries, and this represents active research our team is exploring as a next step toward improving surveillance programs with AI. Our ultimate objective is to have an entirely automated pathway—from data acquisition to summarization and visualization—allowing for data to be collected, summarized, and presented in near-real time. This was once a theoretical possibility in the surveillance space; however, with advancements in NLP and AI, it is likely feasible within the next few years. With this level of high fidelity of data reporting, public health organizations will have both the opportunity and challenge of developing public health interventions that translate into clinical impact. In addition, AI powered reporting systems of this nature may help to assist with the postmarket surveillance of products and pharmaceuticals allowing associated issues to be caught faster while reducing harms to the public.

As AI-based automation begins to make inroads in academic medicine and beyond, impact on jobs and tasks must be considered. As noted in this study, machine learning is able to decrease the time to complete tasks previously done by humans. In our case, automating CHIRPP medical record screening reduces manual workload, freeing coordinators to focus on higher-value tasks such as trends analysis, injury prevention advocacy, and development of public health interventions. This highlights how AI can relieve time-intensive work, allowing humans to engage in more meaningful and strategic activities.

The use of transformer models for patient medical record classification also shows promise for analyzing triage data for other potential use cases. For example, trying to identify certain subsets of patients for research purposes in EDs. If the algorithm is able to identify patients eligible for a given research study in real time, this information can be given directly to study coordinators and improve patient enrollment.

Our research also demonstrates that computationally expensive large language models are not required for prediction tasks such as ours. The use of relatively small transformer models enables research teams and injury surveillance programs to fine tune and securely host highly effective NLP models on local networks. Given the sensitive nature of free-text clinical patient medical records, having the ability to run these algorithms on secure local networks is an attractive option.

### Limitations

One of the limitations of this work is that the NLP algorithm was originally trained on cross-sectional data, which means that data drift and model performance need ongoing re-evaluation with regular intervals of retraining to detect future emerging injury trends. This underscores the importance of ensuring effective machine learning operations frameworks are in place to continuously monitor the model’s performance and automate detecting when performance declines. Models will require retraining to keep up with new injury trends as new products are introduced into the market (ie, hoverboards, e-bikes, and so forth).

## Conclusions

Injury surveillance reporting has typically been completed in a manual fashion, undermining the true potential value of injury surveillance programs. In this prognostic study, NLP transformer models automated aspects of surveillance reporting in our ED for injury and poisoning cases with minimal sacrifices to data capture and sensitivity, resulting in tremendous efficiency. This study has created the foundation for predictive applications that can be expanded to other health promotion and surveillance systems. The COVID-19 pandemic highlighted the need for real-time surveillance data, and expanding this technology will allow artificial intelligence to empower public health research, surveillance, and advocacy at an unprecedented scale and speed.

## Notes

Supplement.
Data Sharing Statement
