# Supplementary material for: AI-Driven Injury Reporting in Pediatric Emergency Departments
Source: JAMA Netw Open. 2025 Jul 31;8(7):e2524154. doi: 10.1001/jamanetworkopen.2025.24154 (PMC12314733; doi:10.1001/jamanetworkopen.2025.24154)
Supplement: Supplement. — Data Sharing Statement [file jamanetwopen-e2524154-s001.pdf]

Data Sharing Statement

Singh. AI-Driven Injury Reporting in Pediatric Emergency Departments. *JAMA Netw Open*. Published July 31, 2025. doi:10.1001/jamanetworkopen.2025.24154

Data
Data available: No

Additional Information
Explanation for why data not available: The study leverages over 200,000 full text patient clinical charts for which we (the research team) do not have permission to share publicly. Specific research collaborations on a case by case basis can be discussed to enable secure and privacy preserving data access as needed (must be deemed appropriate by the data governance committee and research ethics board at the Hospital for Sick Children).
